# 150–200 V Split-Gate Trench Power MOSFETs with Multiple Epitaxial Layers

**DOI:** 10.3390/mi11050504

**Published:** 2020-05-15

**Authors:** Feng-Tso Chien, Zhi-Zhe Wang, Cheng-Li Lin, Tsung-Kuei Kang, Chii-Wen Chen, Hsien-Chin Chiu

**Affiliations:** 1 Department of Electronic Engineering, Feng Chia University, Taichung 407, Taiwan; m0707586@o365.fcu.edu.tw (Z.-Z.W.); clilin@fcu.edu.tw (C.-L.L.); kangtk@fcu.edu.tw (T.-K.K.); 2 Department of Electronic Engineering, Minghsin University of Science and Technology, Hsinchu 304, Taiwan; cwchen@must.edu.tw; 3 Department of Electronic Engineering, Chang Gung University, Taoyuan 333, Taiwan; hcchiu@mail.cgu.edu.tw

**Keywords:** split-gate trench power MOSFET, multiple epitaxial layers, specific on-resistance

## Abstract

A rating voltage of 150 and 200 V split-gate trench (SGT) power metal-oxide- semiconductor field-effect transistor (Power MOSFET) with different epitaxial layers was proposed and studied. In order to reduce the specific on-resistance (R_on,sp_) of a 150 and 200 V SGT power MOSFET, we used a multiple epitaxies (EPIs) structure to design it and compared other single-EPI and double-EPIs devices based on the same fabrication process. We found that the bottom epitaxial (EPI) layer of a double-EPIs structure can be designed to support the breakdown voltage, and the top one can be adjusted to reduce the R_on,sp_. Therefore, the double-EPIs device has more flexibility to achieve a lower R_on,sp_ than the single-EPI one. When the required voltage is over 100 V, the on-state resistance (R_on_) of double-EPIs device is no longer satisfying our expectations. A triple-EPIs structure was designed and studied, to reduce its R_on_, without sacrificing the breakdown voltage. We used an Integrated System Engineering-Technology Computer-Aided Design (ISE-TCAD) simulator to investigate and study the 150 V SGT power MOSFETs with different EPI structures, by modulating the thickness and resistivity of each EPI layer. The simulated R_on,sp_ of a 150 V triple-EPIs device is only 62% and 18.3% of that for the double-EPIs and single-EPI structure, respectively.

## 1. Introduction

Trench power MOSFETs have become a superior device in the medium-to-low voltage power application field. In conventional trench MOSFETs, the gate is isolated from the drain region only by the gate oxide. This results in that trench MOSFETs exhibit large switching losses due to a high gate-to-drain capacitance (C_gd_), which limits its application. In order to reduce the device-switching losses, many studies, such as a thick-bottom oxide layer (TBOX) design, W-gated, and RESURF stepped oxide (RSO) MOSFET, were proposed [1,2,3,4]. All of these structures feather a thick oxide between gate electrodes and drain area, to reduce device C_gd_. The RSO structure uses a thicker oxide at the lower portion of the trench, to reduce C_gd_, while it applies a thinner one at the upper portion of the trench, to be the gate oxide. Because the stepped gate electrode plays a role as an extended field plate (FP) to modulate the electric field (EF) around it, this structure not only reduces the feedback capacitance but also the R_on_, by using a low-resistivity epitaxial layer. Although RSO design can reduce the C_gd_, switching losses are still a big issue when a device is used in a high-frequency application. Split-gate trench (SGT) devices overcame that problem by adding a source electrode located between the gate and drain [5,6,7,8,9]. There are two parts in the trenches for a split-gate structure: The upper electrode is the gate, and the lower one is connected by a separate contact to the source, to play as a field plate to balance the charge in the n^-^ drift epitaxy region. This field plate is surrounded with a thick oxide to be a MOS structure that induces a silicon depletion region once the electrode is biased at a more negative potential than the n^-^ silicon region [10,11,12]. Furthermore, the extended field plate along the drift epitaxy layer shapes the electric field in the drift region that enables the drift depletion area to support a higher drain voltage by using a lower resistivity epitaxy layer to reduce device specific on-resistance [5,13]. In addition, the C_gd_ of an SGT can be reduced significantly because the gate electrodes are shielded from the drain region by these FPs [10,14,15].

Even RSO and SGT power MOSFETs can provide an effective way to reduce device feedback capacitance and R_on_ simultaneously. The on-state resistance for a device used in a higher voltage system (100 to 200 V) increases sharply, owing to a high-resistivity epitaxial layer. For 20–30 V low-voltage SGT devices, the channel resistance portion is dominant and amounts to over 60%–85% of the total device resistance. However, this channel resistance is reduced to only 30%–20% for 60–70 V middle-voltage-rating devices [16,17]. When the device rating voltage reaches 150–200 V, the drift resistance occupies about 90% of the total device resistance [16,18]. To achieve a high breakdown voltage (V_BR_) design without increasing the R_on_ too much, a gradient, two-stepped oxide or multiple stepped oxide designs were applied to the trenches and shown to improve device performance effectively [18,19,20,21]. Since the potential of the field plate (bottom gate) on the oxide around it is different everywhere, that leads to a different depletion strength and electric field between two trenches along the cell depth, [18,19,20,21] use oxide engineering to improve device performance. On the other hand, double split-gate resurf stepped oxide UMOS can overcome the non-uniform problem [15]; however, the oxide and poly process in the trenches is too complicated. The abovementioned methods could make the drift region have a more uniform EF distribution to sustain a higher V_BR_. However, these structures required multiple depositions and etching steps that complicate the fabrication process. Superjunction structures and wide bandgap SiC material devices are alternative ways to provide high-voltage and low-R_on,sp_ solutions [22,23,24,25]. However, the built-in superjunction depletion layer limits the scalability to lower voltages (<500 V) [3]. In addition, besides cost issues, low channel mobility owing to a high density of SiC/SiO_2_ interface traps and undesirable higher turn on voltage of the body diode of a wide bandgap SiC power MOSFET make SiC devices less attractive than Si ones for lower-voltage applications [26,27,28]. Lower-voltage SiC power MOSFETs have not yet been demonstrated [10]. For a device structure with a rating voltage below 200 V, Si SGT power MOSFET dominates and plays an important role in reducing the device R_on,sp_ in power applications.

In this study, we proposed a 150 V SGT power MOSFET with multiple EPIs, to improve the device characteristics, and applied the same way to design a 200 V SGT power device. The single-EPI structures are wildly used in the low-voltage (<50 V) SGT power MOSFETs design. When required device rating voltage is up to 50–100 V, single-EPI device makes this scheme suffer a sharply increased R_on_. A double-EPI-layers structure was used to improve device R_on_ characteristics in some studies [29,30]. Compared to the single-EPI one, the double-EPIs device has a higher device output current than the single one. This unique merit allows for the possibility of the double-EPIs design to reduce R_on,sp_, as well as its power consumption. In this study, we wanted to design and modify the EPI structures rather than the complicated fabrication ways mentioned in [15,18,19,20,21], to reduce device R_on_ and sustain a high V_BR_ at the same time. When device ranting voltage is designed to over 100 V, we find that the R_on_ of double-EPIs structure is no longer satisfying our expectations. Therefore, a triple-EPIs structure was applied, to modify the EF distributions between two trenches, instead of only depending on its magnitude supported by the bottom EPI. This design makes us have more flexibilities in designing the bottom EPI with a lower resistivity specification, to achieve a lower R_on,sp_ device. In double-EPIs design, the bottom EPI layer is used to support the V_BR_, and the top one could be used to modify the EF and reduce the R_on_. For a triple-EPIs structure, the top and bottom EPI layers play the same roles as those is the double-EPIs device. The middle one is used to lower the R_on,sp_ if the top and bottom EPI layers can be properly designed. We applied ISE-TCAD to simulate and investigate by analyzing device potential and EF distributions with different epitaxial layers for all devices [31]. The R_on,sp_ of a triple-epitaxial-layer structure is much lower than those applied with a single- or double-epitaxial layer based on the same fabrication process.

## 2. Device Structure and Simulation

**Figure 1:**
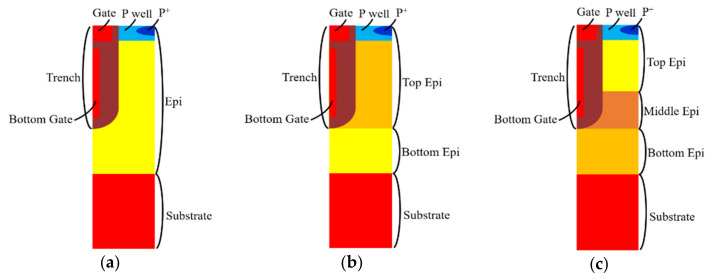
Split-gate trench power MOSFET structure with (**a**) single EPI, (**b**) double EPIs, and (**c**) triple EPIs.

**Figure 2:**
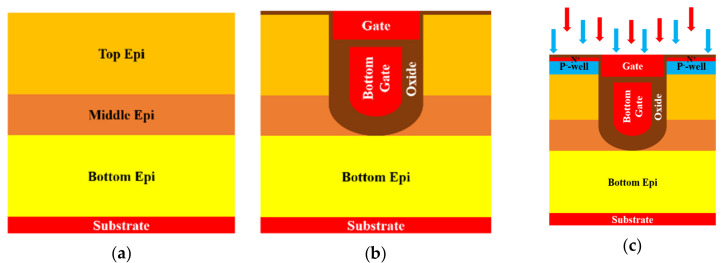

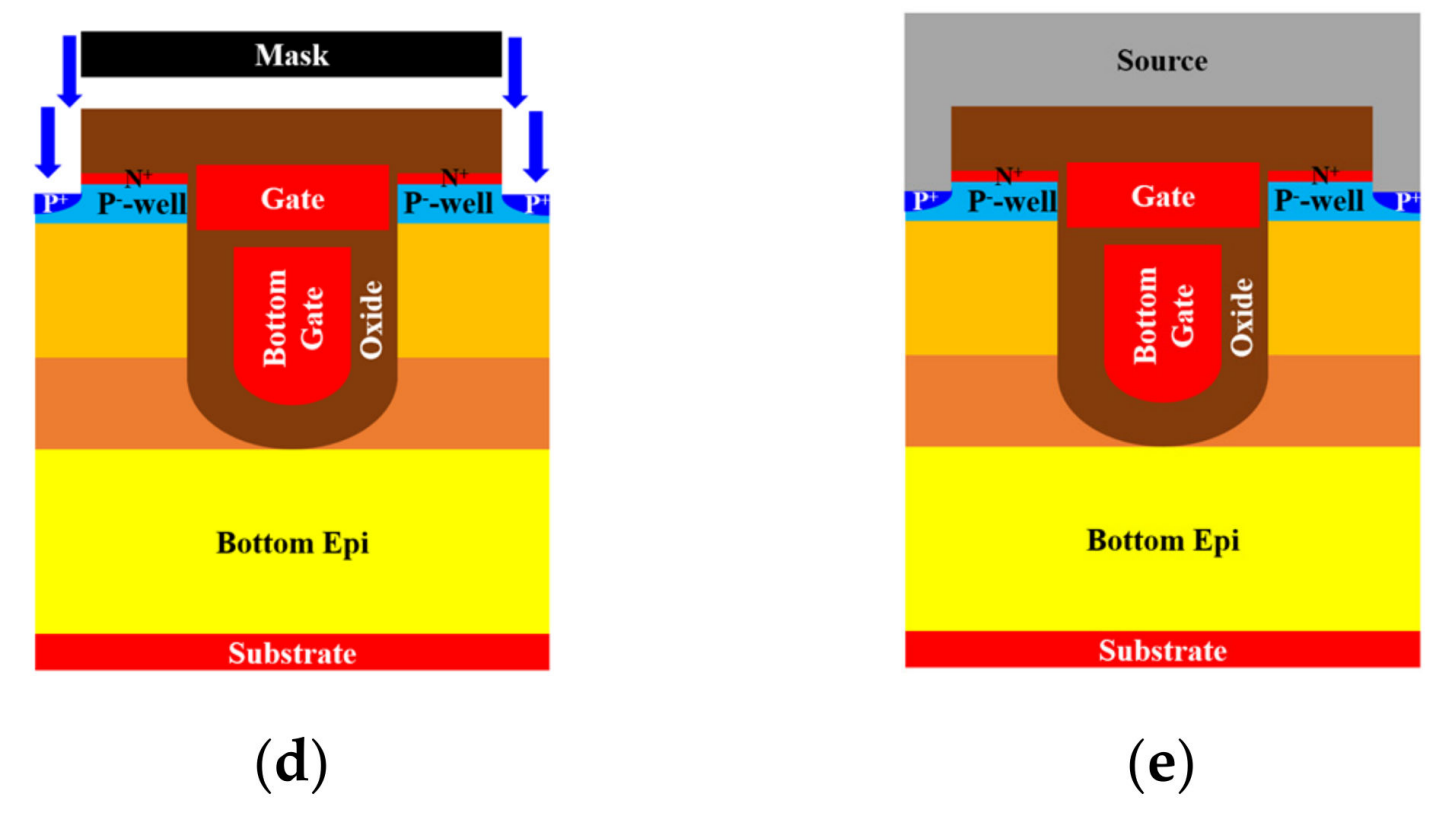
Main simulated fabrication process for the triple-EPIs SGT power MOSFET: (**a**) three designed epitaxial layers; (**b**) defining the trench, bottom gate, and gate; (**c**) forming the channel and the source areas; (**d**) opening the contact holes and implanting the p^+^; (**e**) depositing the metal pads.

**Figure 3:**
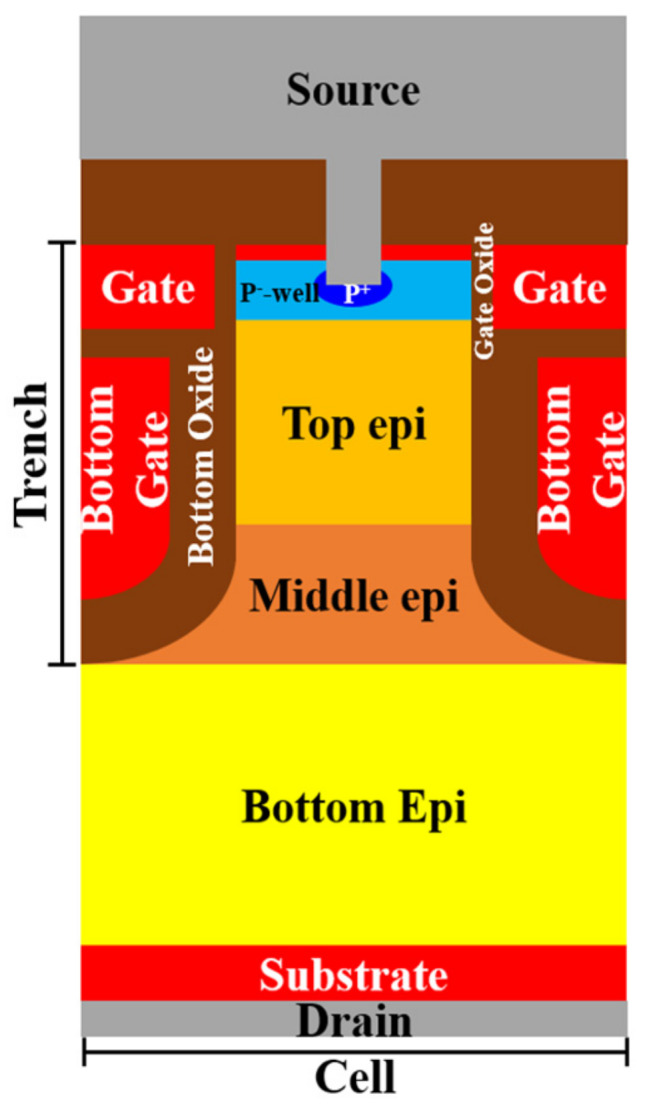
The cross-section diagram of a triple-EPIs SGT power MOSFET.

Multiple EPI structures were applied in this study. Figure 1 shows the trench location related to each structure with different epitaxial layers designs. The process steps of simulation for a three-epitaxial-layer device are shown in Figure 2. We started with a designed three EPIs above an n^+^ substrate. Detailed layers’ information is listed in Table 1. A trench was first defined and etched to the top of the bottom EPI. An oxide and polysilicon (Poly-Si) were sequentially deposited. After that, the deposited Poly-Si was etched back, to form a bottom gate. Then, the gate oxide was grown thermally, and a Poly-Si layer was deposited to fill the trenches and then etched to play the gates. Next, the device was implanted to accomplish the p^-^-well and n^+^-well as the channel and the source region, respectively. After an oxide was deposited and contact holes were opened, an etching process was applied, and a p^+^ implantation was employed to improve the device’s ruggedness. Finally, a metal was formed to be the source electrode. Figure 3 shows the cross-section of this SGT power MOSFET structure with three epitaxial layers.

## 3. Results and Discussion

**Figure 4:**
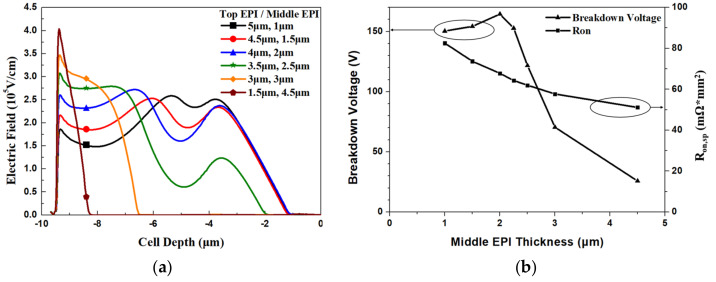
(**a**) The simulated electric field curves in the middle of the cell and (**b**) the simulated V_BR_ and R_on,sp_ with different top- and middle-EPI-thickness designs with the same total and bottom EPI thickness.

First, we constructed a 150 V device by using a double-EPIs structure. The trench depth we used here was 6 µm, from the top to the bottom EPI. For a double-EPIs structure, to improve its V_BR_, a thicker thickness or a higher-resistivity bottom EPI is required. However, it will increase R_on_ significantly. Then, we apply the same EPI thickness and trench depth as double-EPIs structure to all devices in the simulation. For comparison, we adopted the same bottom EPI specification for the double structure used as for the single-EPI device. For a triple-EPIs device, the EPI specifications are adjusted to achieve a balance to have a maximum V_BR_ and a minimum R_on,sp_. The EPI information for all structures is list in Table 2. All the devices simulated here use the same trench depth (6 µm). Different top- and middle-EPI-thickness designs are studied for a triple-EPIs device. Figure 4a shows the EF distributions with different top- and middle-EPI-thickness designs. We can see that the EF distributions between two trenches can be modified by different top and middle EPI thickness. Our approach to improving the EF distributions between two trenches is similar to that proposed in [15]. We used triple EPIs and [15] double split-gates with different bias in the trenches, to achieve the same purpose. The R_on_ is not affected by the top EPI too much; however, different electric field distributions with different EPI combinations here give us more room to design a high V_BR_ device. One can expect that the highest breakdown voltage can be obtained in the largest area of the EF integration, with respect to the cell depth [19,22]. In our study, the best top-and middle-EPI-thickness ratio to sustain a high V_BR_ device is 1:2. Figure 4b presents the simulated V_BR_ and R_on,sp_ with different EPI-thickness designs.

**Figure 5:**
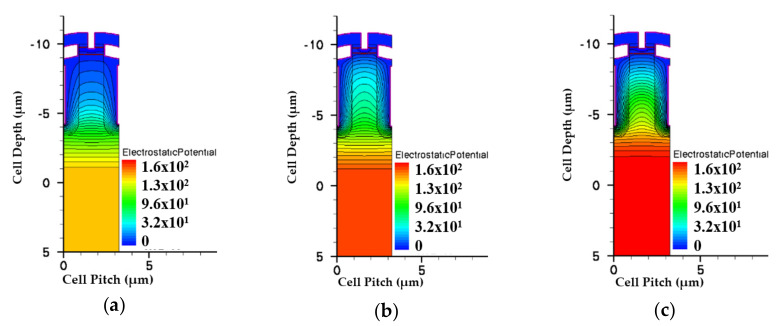
The simulated potentials for the split-gate trench power MOSFET with (**a**) single EPI, (**b**) double EPIs, and (**c**) triple EPIs at the same EPI thickness. The color bars are scaled on the same degree.

**Figure 6:**
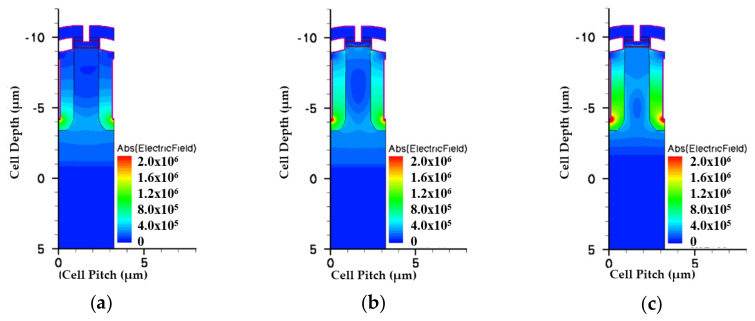
The simulated electric fields for the split-gate trench power MOSFET with (**a**) single EPI, (**b**) double EPIs, and (**c**) triple EPIs at the same EPI thickness. The color bars are scaled on the same degree.

**Figure 7:**
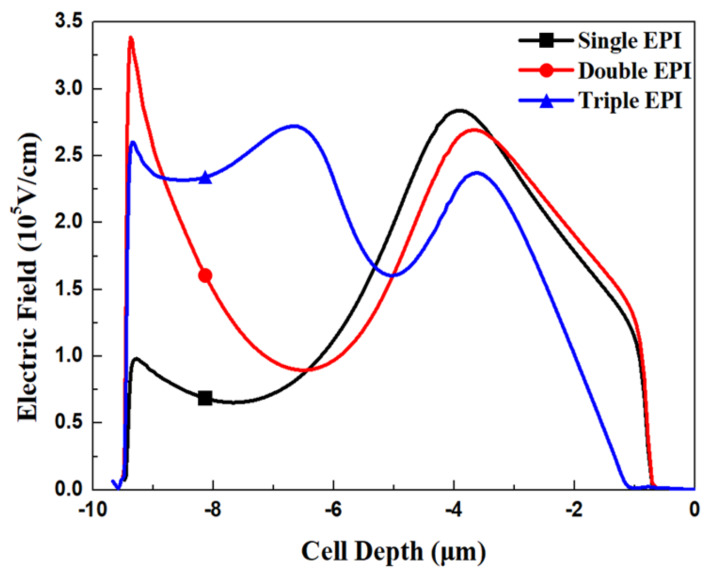
The simulated electric field curves for all devices with single EPI, double EPIs, and triple EPIs with the same total EPI thickness.

Figure 5 and Figure 6 show the simulated potential and EF distributions for all structures under the same total EPI thickness. From the simulation, it is obvious that the triple-EPIs device can sustain a higher V_BR_ easily than the others. A middle EPI layer is used to increase the EF magnitudes and then enhance the breakdown, as well as lower the R_on,sp_ simultaneously. In addition, it offers us more flexibility to adjust the resistivity of the bottom EPI, to further reduce its R_on,sp._ The EF distribution curves in the cell center for all structures are shown in Figure 7. From this figure, the triple-EPIs design shows it has more uniform EF distributions between two trenches to sustain a higher V_BR_. In Figure 7, it is obvious that the two-layer structure can increase the device top electric field between two trenches; however, it decreases to a low value at p^-^-well/n^-^EPI as the single one does. A triple-EPIs structure is designed to enhance the device EF between two trenches at the top and p^-^ well/n^−^ EPI area between two trenches to enhance device breakdown voltage. From Figure 7, we can observe a more uniform electric field distribution and the largest area under EF integration along the cell depth can be found in the triple-EPIs design. Therefore, the breakdown voltage of a triple-EPIs device can be improved. All devices’ performances are summarized in Table 3. By using the same EPI thickness, the triple-EPIs design has the highest breakdown voltage than other EPI structures. 

**Figure 8:**
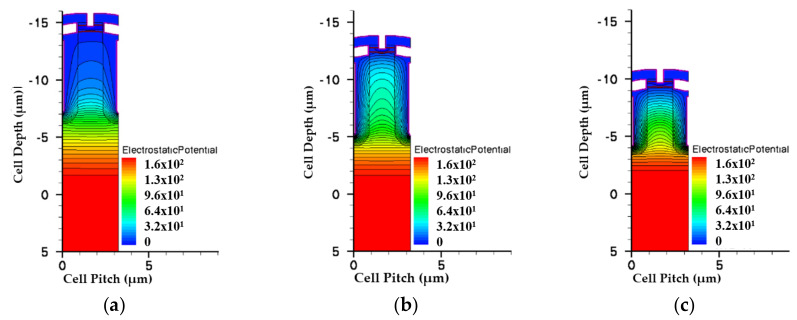
The simulated potentials for the split-gate trench power MOSFET with (**a**) single EPI, (**b**) double EPIs, and (**c**) triple EPIs at 150 V rating voltage. The color bars are scaled on the same degree.

**Figure 9:**
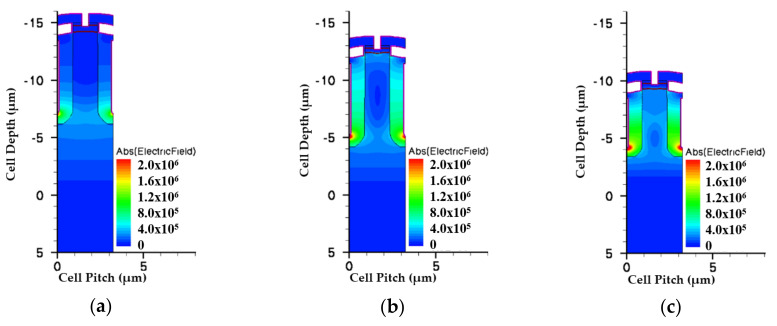
The simulated electric fields for the split-gate trench power MOSFET with (**a**) single EPI, (**b**) double EPIs, and (**c**) triple EPIs at 150 V rating voltage. The color bars are scaled on the same degree.

**Figure 10:**
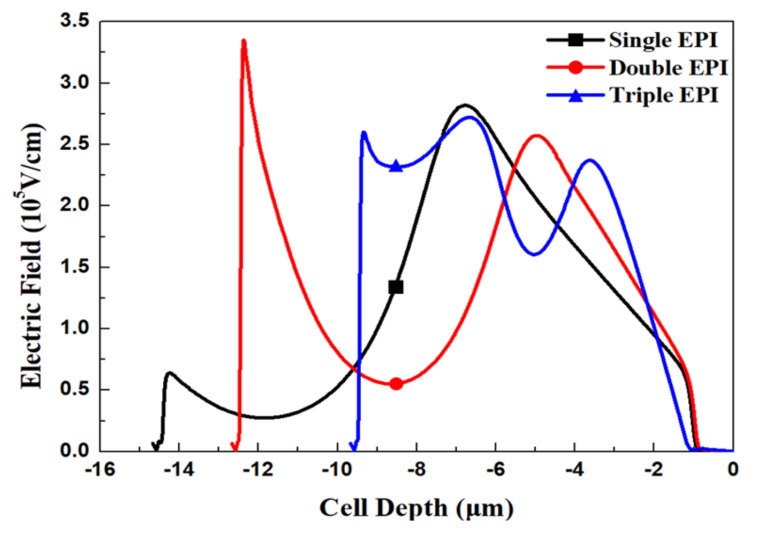
The simulated electric field curves for the split-gate trench power MOSFET with single EPI, double EPIs, and triple EPIs at 150 V rating voltage.

Then we modified single-EPI and double-EPIs specifications to sustain the same V_BR_ that a triple design can achieve. To increase the V_BR_ of these two devices, the thickness and resistance of each EPI layer, as well as the trench depth, have to be increased. The EPI information for all structures is list in Table 4. Figure 8 shows the potential profiles for all devices. It can be seen that, in order to sustain a higher rating voltage, the thickness and resistivity of the single-EPI and double-EPIs structure must be thickened and increased to achieve a high V_BR_. The EF magnitude distributions of all structures are shown in Figure 9 and Figure 10. We can find that, the less EPI layers that are used, the lower the electric field valley, which weakens the support of a high V_BR_ with a small R_on,sp_. The triple-EPIs structure uses a middle EPI to enhance its electric field in the middle of the trench, where there is an EF valley observed in other structures. Therefore, a triple-EPIs structure is much easy to sustain a high V_BR_ than other devices.

**Figure 11:**
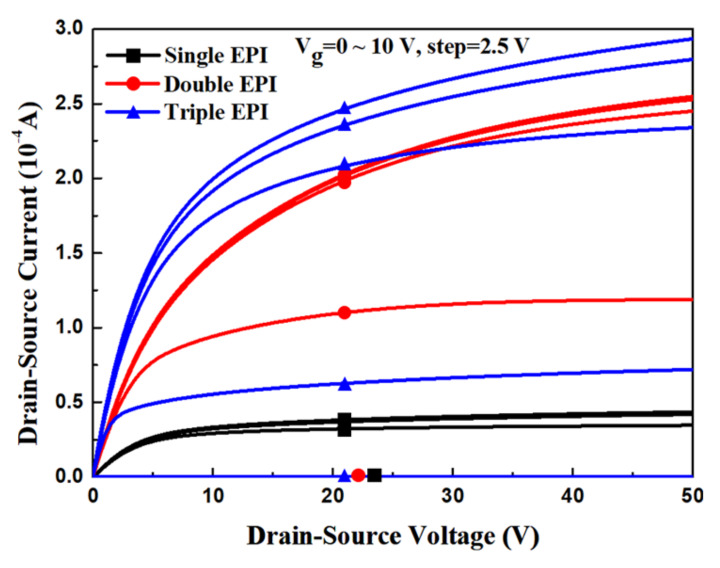
The output characteristic of the split-gate trench power MOSFET with single EPI, double EPIs, and triple EPIs.

Figure 11 shows the output characteristics for all structures with the same V_BR_ of 164 V. It can be observed that the R_on_ of a triple-EPIs design is much lower than those of the others. The triple-EPIs structure can sustain a higher V_BR_, owing to a more uniform electric field distribution between two trenches that is attributed to top- and middle-EPI design. It makes a triple-EPIs device more flexible on resistivity and thickness design for bottom EPI to achieve a low R_on_ characteristic. Table 5 demonstrates the R_on,sp_ for all devices with the same V_BR_. The simulated R_on,sp_ of a triple-EPIs device with a rating voltage of 150 V is only 62% and 18.3% of the one for the double-EPIs and single-EPI structure, respectively. Although a double-EPIs structure has better R_on,sp_ than the single one, the long trench depth, accompanied by a long top EPI thickness, makes it is hard to maintain a uniform electric field between two trenches. Therefore, a higher resistivity bottom EPI spec is required to sustain a high rating voltage that results in a higher R_on_ than the triple-EPIs design. Compared with other methods mentioned in [15,18,19,20,21], the multiple-EPIs structure does not complicate the process in manufacturing, and a higher-V_BR_ and a lower-R_on,sp_ device can be achieved.

We also use the same method to construct 200 V SGT devices with different EPI designs. Similar electrical field distributions and output characteristics with Figure 10 and Figure 11 can be obtained, respectively, if we modify the best epitaxial specification. Table 6 lists the parameters that we used for all 200 V SGT devices’ simulation and shows their characteristics. Again, the triple-EPIs structure demonstrates more flexibility to achieve a lower R_on,sp_ than the single-EPI and double-EPIs devices under the same breakdown voltage design.

**Figure 12:**
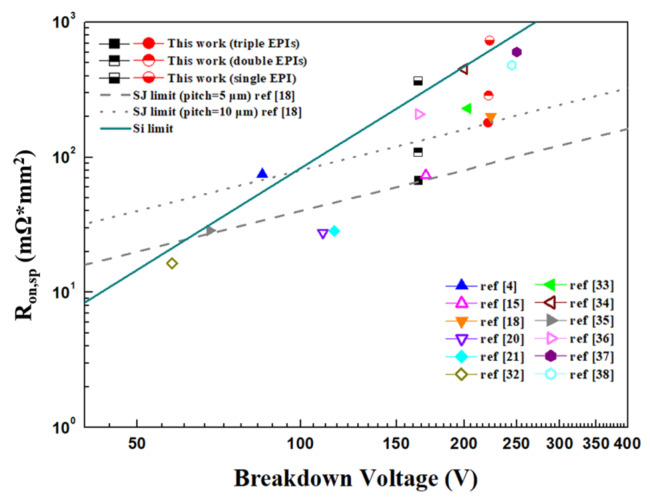
The comparison of R_on,sp_ against V_BR_ relationship of middle-voltage SGT structures, super junction devices, ideal silicon limit and super junction (SJ) limit (cell pitch = 5 and 10 µm).

Figure 12 compares the specific on-resistance performance of our proposed SGT devices with that of the other middle-voltage devices reported in [4,15,21,32,33,34,35,36,37,38,39,40], ideal silicon limit, and super junction (SJ) limit for cell pitch = 5 and 10 µm in the 50–200 V range. Form Figure 12, we observe that the triple-EPIs structure and those using a double split-gate device [15] and stepped oxide SGTs [18,20,21] can achieve a very low R_on,sp_ in the middle-voltage range because they all can maintain more uniform EF distributions between two trenches. Compared with a double split-gate device and stepped oxide ones, our triple-EPIs devices do not require the complicated double split-gate or oxide-engineering process in the trenches and is compatible with the conventional SGT process.

## 4. Conclusions

A 150–200 V rating voltage triple-EPIs SGT-power MOSFET was proposed, studied, and compared with a single-EPI and double-EPIs structure. The middle EPI in the triple-EPIs structure is used to increase the low electric field between two trenches, thereby increasing the breakdown voltage and reducing the on-resistance. Compared with the single-EPI and double-EPIs structures, the triple-EPIs SGT-power MOSFET had a lower on-resistance. The simulated R_on,sp_ of a triple-EPIs device with a rating voltage of 150 V is only 62% and 18.3% of the one for the double-EPIs and single-EPI structure, respectively.

## Figures and Tables

**Table 1:** The simulation parameters that were used for the triple-EPIs structure.

Parameter	Value
Cell pitch	3.24 µm
Thickness of top EPI	4 µm
Resistance of top EPI	0.9 Ω·cm
Thickness of middle EPI	2 µm
Resistance of middle EPI	0.16 Ω·cm
Thickness of bottom EPI	4 µm
Resistance of bottom EPI	0.68 Ω·cm
Depth of trench	6 µm
Width of trench	1.8 µm
Thickness of bottom oxide	0.8 µm
Thickness of gate oxide	0.06 µm

**Table 2:** The parameters that were used for the single-, double-, and triple-EPI structure simulation.

Device	EPI Thickness (µm)	EPI Resistance (Ω·cm)
Single EPI	10	1.4
Double EPIs	Top EPI = 6	Top EPI = 0.35
	Bottom EPI = 4	Bottom EPI = 1.4
Triple EPIs	Top EPI = 4	Top EPI = 0.9
	Middle EPI = 2	Middle EPI = 0.16
	Bottom EPI = 4	Bottom EPI = 0.68

**Table 3:** The characteristics of single-EPI, double-EPIs, and triple-EPIs SGT power MOSFET with the same total EPI thickness.

Device	Breakdown Voltage (V)	R_on_ (mΩ·mm^2^)	EPI Thickness (µm)
Single EPI	130.93	181.16	10
Double EPIs	153.85	98.23	10
Triple EPIs	164.49	67.79	10

**Table 4:** The parameters that were used for the single-EPI, double-EPIs, and triple-EPIs structure simulation.

Device	EPI Thickness (µm)	EPI Resistance (Ω·cm)	Trench Depth (µm)
Single EPI	15	2	8
Double EPIs	Top EPI = 9	Top EPI = 0.35	8
	Bottom EPI = 4	Bottom EPI = 1.4	
Triple EPIs	Top EPI = 4	Top EPI = 0.9	6
	Middle EPI = 2	Middle EPI = 0.16	
	Bottom EPI = 4	Bottom EPI = 0.68	

**Table 5:** The characteristic of single-EPI, double-EPIs, and triple-EPIs 150 V rating voltage split-gate trench power MOSFET at the same cell pitch.

Device	Breakdown Voltage (V)	R_on_ (mΩ·mm^2^)	Cell Pitch (µm)
Single EPI	164.2	369.85	3.24
Double EPIs	164.23	109.56	3.24
Triple EPIs	164.49	67.79	3.24

**Table 6:** The parameters that were used for the single-EPI, double-EPIs, and triple-EPIs structure simulation.

Device	EPI Thickness (µm)	EPI Resistance (Ω·cm)	Breakdown Voltage (V)	R_on_ (mΩ·mm^2^)
Single EPI	16	4.8	222.79	729.7
Double EPIs	Top EPI = 6	Top EPI = 0.3	221.73	286.47
	Bottom EPI = 9	Bottom EPI = 3		
Triple EPIs	Top EPI = 4	Top EPI = 0.8	221.33	184.36
	Middle EPI = 2	Middle EPI = 0.17		
	Bottom EPI = 7.5	Bottom EPI = 2.1		
